# Antifungal Activities of Phytochemically Characterized Hydroethanolic Extracts of *Sclerocarya birrea* Leaves and Stem Bark against Fluconazole-Resistant *Candida albicans* Strains

**DOI:** 10.1155/2022/4261741

**Published:** 2022-06-07

**Authors:** Benjamin Kingsley Harley, David Neglo, Mike Okweesi Aggrey, Anthony Martin Quagraine, Emmanuel Orman, Jonathan Jato, Nana Ama Mireku-Gyimah, Cedric Dzidzor K. Amengor, Theophilus Christian Fleischer

**Affiliations:** ^1^Department of Pharmacognosy and Herbal Medicine, School of Pharmacy, University of Health and Allied Sciences, Ho, Ghana; ^2^Department of Basic Science, School of Basic and Biomedical Sciences, University of Health and Allied Sciences, Ho, Ghana; ^3^Department of Pharmaceutical Chemistry, School of Pharmacy, University of Health and Allied Sciences, Ho, Ghana; ^4^Department of Pharmacognosy and Herbal Medicine, School of Pharmacy, University of Ghana, Accra, Ghana

## Abstract

The study evaluated the antifungal activities of the 70% ethanol extracts of *Sclerocarya birrea* leaves (SBL) and stem bark (SBB) against *C. albicans* strains and fluconazole-resistant isolates, their antifungal effects in combination with conventional antifungals as well as their effects on the biofilms of the *C. albicans* strains and isolates. UPLC-QTOF-MS/MS analysis was then carried out to investigate the metabolite profile of the extracts and UPLC fingerprints developed for their routine identification as part of quality control measures. The extracts exhibited considerable antifungal activity with MIC ranging from 12.21 to 97.66 *μ*g/mL and MFC from 12.21 to 390.63 *μ*g/mL against the *C. albicans* strains and isolates. The antifungal activity of the stem bark extract was higher than the leaf extract. SBL and SBB also significantly inhibited biofilm formation (IC_50_ = 12.49 to 164.42 *μ*g/mL) and the mature biofilms (IC_50_ = 91.50 to 685.20 *μ*g/mL) of the strains and isolates of the *C. albicans* and demonstrated potential for their use in combination therapies with currently used antifungals especially the stem bark extract with nystatin. Metabolite profiling identified the presence of polyphenolic compounds in both leaves and stem bark mostly flavonoids, their derivatives, and proanthocyanidins, which contribute in part to the bioactivity of the plant. Whereas flavonoids like quercetin, myricetin, and their derivatives were abundant in the leaves, epicatechin monomers with their condensed tannins, including procyanidin B2 and procyanidin C, were abundant in the stem bark. Fingerprints of SBL and SBB were developed and validated and could be used as qualitative tools to authenticate the plant. The outcomes of the study show the promise of the leaf and stem bark extracts of *S. birrea* to be studied further and developed as antifungal agents.

## 1. Introduction

Vulvovaginal candidiasis (VVC), the second most common vaginal infection among women of reproductive age, is a disease caused by fungi of the genus *Candida* [1]. Surveys suggest that about 70-75% of women develop vulvovaginal candidiasis once in their lifetime [2] with *Candida albicans*, the causative organism in majority of the cases [3], although candidiasis caused by non-*C. albicans* species especially *C. glabrata* and *C. auris* has been on the ascendancy in recent times [4]. *Candida albicans* is a component of the normal vaginal flora and only becomes an opportunistic pathogen that causes VVC when there is a decline in the body's immunity [5]. VVC causes a lot of physical and emotional discomforts in patients, poses a challenge to healthcare providers, and produces considerable financial strain on patients and their caregivers due to medication costs and hospital visits [6]. Coupled to these untoward implications, the disease has been associated with high risk of complications in pregnancy including congenital cutaneous candidiasis, candida chorioamnionitis, preterm delivery, and abortion [7].

Currently, there are no approved immunotherapies or vaccines against fungal infections. The typical first-line chemotherapy for VVC is with azole-class imidazole and triazole antifungals which can be administered topically or orally [8]. Other commonly used antifungals include the polyenes, mainly nystatin and echinocandins like caspofungin [9]. Although most antifungal medications are available, the choice of treatment by patients especially in sub-Saharan Africa is influenced by their socioeconomic conditions. These oral antifungal medications also possess adverse effects such as gastrointestinal disorders and headaches whereas the topical agents are known to cause hypersensitivity reactions like burning, itching, and erythema [10]. Over the past few years, fluconazole has emerged as the drug of choice for VVC owing to its efficacy, safety, favourable pharmacokinetics, and availability as a generic product [11]. Its extensive use for both acute episodes and maintenance regimen has resulted in fluconazole resistance in the causative organisms particularly *C. albicans* due to the over exposure of the agent to the *Candida* species [12]. Similarly, there has been an upward trend in resistance across the various classes of antifungals in the *Candida* species lately [13]. This emergence of drug-resistant fungi poses a major threat to human health and underscores the urgent demand for the discovery and development of new antifungal agents.

Traditional medicine practice is widely employed by a large populace of the world especially in developing countries for their health and well-being, with medicinal plants as the agents of choice in most traditional medicine practices in treating both infectious and non-infectious diseases [14]. One of such plants used in the treatment of communicable diseases among the indigenous Ghanaian population is *Sclerocarya birrea* (A. Rich.) Hochst (family: Anacardiaceae). It is a tree usually located in the semi-arid, deciduous, and savannah areas of sub-Saharan Africa and grows in wooded grasslands and bushlands [15]. *S. birrea* is widely distributed across West and East Africa from Gambia to Sudan [16]. The plant is a single-stemmed, perennial, medium-sized tree that grows up to 13 meters high with stout branchlets and grey fissured bark [17]. Its compound leaves possess 7–13 pairs of leaflets together with a terminal one, crowded near the end of the branches. The leaf is about 60 mm long that tapers abruptly into a narrow end with a dark-green upper surface but lighter underneath [18]. *S. birrea* bears highly aromatic and fleshy fruits with sweet-sour taste which has become part of the diet of Southern Africa [19].

Various parts of the plant are used extensively in traditional medicine for treating several ailments. The leaves and fruits are used in treating coughs, diabetes, dysentery, scorpion and snake bites, malaria, inflammations, and hypertension [20]. The stem bark decoctions of the plant are taken to treat diarrhoea and dysentery and administered to patients suffering from gangrenous rectitis [21]. *S. birrea* stem bark is also utilized by South Africans in treating fevers and ulcers whereas the roots are used in the treatment of sore eyes, pharyngitis, goitre, and splenomegaly [22]. Due to the plethora of applications of *S. birrea* in folk medicine, extracts of the plant have been studied and shown to possess antidiabetic [23], antihypertensive [24], antibacterial [25], antiparasitic [26], anti-inflammatory [27], and antioxidant [28] activities. Previous phytochemical investigation of the stem bark of *S. birrea* led to the isolation of quercetin derivatives such as quercetin 3-O-*α*-(5′′-galloyl) arabinofuranoside, quercetin 3-O-*β*-D-(6′′-galloyl) glucopyranoside, quercetin 3-O-*β*-D-glucopyranoside, quercetin 3-O-*α*-L-rhamnopyranoside, and quercetin 3-O-*β*-D-(6′′-galloyl)galactopyranoside; together with other phenolic substances including gallic acid, kaempferol 3-O-*β*-D-(6′′-galloyl) glucopyranoside, (-)-epicatechin 3-O-galloyl ester, kaempferol 3-O-*α*-L-rhamnopyranoside, myricetin 3-O-*α*-L-rhamnopyranoside, and (-)-epigallocatechin 3-O-galloyl ester [20]. The presence of some of these polyphenolic compounds has also been confirmed through HPLC-MS analysis of extracts of the plant. Whereas flavonoid glycosides and galloylated glycosides of quercetin and kaempferol are prominent in the leaf extracts, galloylated tannins and procyanidins are present in the stem and root barks [29–32].

Roots of *S. birrea* are a major component of some antifungal herbal products used in the treatment of candidiasis on the Ghanaian markets. Previous investigations have demonstrated the potent antifungal activity of the ethanolic root extracts of *S. birrea* against susceptible *Candida* species [33]. However, the use of the roots of the plant by the herbal industry, who are dependent on the continuous supply of raw materials, is unsustainable and could lead to extinction. Additionally, there is no reported study on the antifungal activity of the plant against resistant strains of the *Candida* species. Furthermore, the development of chromatographic fingerprints for the unequivocal identification and quality assessment of *S. birrea* is of paramount importance in the face of adulteration. Consequently, this study sought to investigate the antifungal activity of the more sustainable leaves and stem bark of *S. birrea* against fluconazole-resistant isolates of *C. albicans*, assess their inhibitory activity on the biofilms of the resistant *C. albicans* isolates, and evaluate their effects on the activity of conventional antifungal agents. UPLC-QTOF-MS/MS analysis was carried out to characterize the bioactive phytoconstituents in the extracts and UPLC fingerprints for the purposes of routine identification as part of quality control measures of the leaves and stem bark are also reported.

## 2. Materials and Methods

### 2.1. Chemicals

Fluconazole, Mueller-Hinton (MH) agar, voriconazole, Sabouraud Dextrose Agar (SDA), and chloramphenicol were bought from Thermo Fisher (Oxoid Limited, Hampshire, UK). Nystatin and caspofungin were bought from Sigma Aldrich (St. Louis, MO, United States).

### 2.2. Plant Collection, Processing, and Extraction

The leaves and stem bark of *S. birrea* were collected in the Savanna areas of Wa, Upper West Region of Ghana, in July 2020 during the dry season and identified by Mr. Alfred Ofori at the Institute of Traditional and Alternative Medicine (ITAM), University of Health and Allied Sciences (UHAS), where voucher specimens have been deposited (Voucher specimen numbers: UHAS/ITAM/2020/L004 and UHAS/ITAM/2020/SB005 for the leaves and stem bark, respectively). The plant materials were then cleaned thoroughly, chopped into pieces, and thereafter air-dried for 7 days before grinding into coarse powders.

Powdered leaves and stem bark of *S. birrea* (500 g each) were cold macerated separately with 70% hydro-ethanol (3 x 3 days) at room temperature amid constant shaking with a mechanical shaker. The extracts obtained were combined and lyophilized into solid extracts with a Lyotrap-Ultra freeze dryer (LTE Scientific, United States) [34]. The weight of the extracts obtained was 32.75 g (yield: 6.55%) and 23.39 g (yield: 4.68%) and designated as SBL (leaves) and SBB (stem bark), respectively.

### 2.3. Antifungal Testing

#### 2.3.1. Fungal Strains and Growth Conditions

Reference *Candida albicans* strains ATCC 90028, ATCC 10231, and SC5314 were obtained from Thermo Scientific™ (Waltham, MA USA).

*C. albicans* clinical isolates from pregnant women with VVC were sourced from the Microbiology Laboratory Department, Ho Teaching Hospital, Ghana. Separate fungal colonies were subcultured on SDA supplemented with chloramphenicol and incubated at 37°C for 48 h to ensure pure *Candida* isolates were obtained. The *C. albicans* isolates were identified using HiCrome Candida Differential Agar (HiMedia Laboratories, India). Isolates were incubated at 35°C for 48 h, and the colours produced were recorded. C. albicans isolates were identified by their smooth and light green colours. Confirmation was carried out using API ID 32C strips (Biomerieux, France) [35].

#### 2.3.2. Fluconazole Susceptibility Test of the C. albicans Isolates

The sensitivity of the *C. albicans* isolates against fluconazole (25 *μ*g) or otherwise was investigated using a disc diffusion method [36] with slight modifications. In brief, isolates of *C. albicans* from the SDA plates were emulsified with 0.85% sterile saline solution to obtain a suspension of turbidity 0.5 McFarland. Media lawns were then seeded in three dimensions with sterile swabs dipped in the prepared suspension. Fluconazole-loaded disks were thereafter placed on the lawn aseptically before incubating for 24-48 h at 37°C. Zone diameters produced by the fluconazole disks were measured using a ruler. Zone diameters of ≥19 mm were deemed susceptible, 15 to 18 mm dose-dependently sensitive, and ≤14 mm resistant. As such, five fluconazole-resistant *C. albicans* isolates were selected for study and were assigned as CA-R1, CA-R2, CA-R3, CA-R4, and CA-R5, respectively.

#### 2.3.3. Evaluation of Antifungal Activity

The antifungal effect of the 70% ethanol extracts of *S. birrea* leaves (SBL) and stem bark (SBB) was evaluated using the broth microdilution method in accordance with document M27-A3 by the Clinical and Laboratory Standards Institute (CLSI) (2008) [37] with slight modifications. Voriconazole and fluconazole were used as positive controls and the blank media employed as negative control. The Minimum Inhibitory Concentrations (MICs) were determined visually before confirmation using spectrophotometry at 490 nm using a microplate reader. Each experiment was carried out in triplicate.

The antifungal activities of the extracts were interpreted as follows: very strong bioactivity, <3.52 *μ*g/mL; strong bioactivity, 3.52–25 *μ*g/mL; moderate bioactivity, 26–100 *μ*g/mL; weak bioactivity, 101–500 *μ*g/mL; very weak bioactivity, 501–2000 *μ*g/mL; and no activity, >2000 *μ*g/mL [38].

#### 2.3.4. Determination of Minimum Fungicidal Concentration (MFC)

To assess the fungicidal effect of the extracts, aliquots from each well from the antifungal activity assay were transferred onto SDA plates which were thereafter incubated for 48 h at 37°C. The plates were then analyzed for the presence or absence of growth [39].

### 2.4. Effect of the Extracts in Combination with Clinically Used Antifungals

The antifungal effect of SBL and SBB when combined with fluconazole, nystatin, or caspofungin, respectively, were determined using the checkerboard assay modified from EUCAST-AFST guidelines reference technique [40]. MICs were determined by visually before confirmation using spectrophotometry at 490 nm using a microplate reader. Each experiment was carried out in triplicate.

The result was analyzed by calculating the Fraction Inhibitory Concentration Indices (FICI) which is a nonparametric model built on the Loewe additivity theory. FICI was determined as follows: FICI = FIC_A_ + FIC_B_, where FIC_A_ = (MIC_CA_/MIC_A_) and FIC_B_ = (MIC_CB_/MIC_B_). MIC_A_ and MIC_B_ are the Minimum Inhibitory Concentrations (MIC) of A and B alone; and MIC_CA_ and MIC_CB_ are the Minimum Inhibitory Concentrations of A and B when used in combination.

The FIC Indices were interpreted as follows: synergism (FICI ≤ 0.5), indifference (>0.5-4.0), and antagonism (>4.0) [41].

### 2.5. Activity of the Extracts against *C. albicans* Biofilms

The inhibition of biofilm formation and activity against preformed biofilms of the *C. albicans* strains of SBL and SBB were determined using the 96-well plates according to methods described below.

#### 2.5.1. Inhibition of Biofilm Formation Assay

Briefly, 50 *μ*L of RPMI 1640 was pipetted into wells of a 96-well microplate together with 50 *μ*L of the extracts in column 1. This was serially diluted till column 10 to obtain concentrations 1000–3.91 *μ*g/mL. Thereafter, 50 *μ*L of fungal inoculum at concentration of 2 × 10^6^ cells/mL was added to well of columns 1–11 of the plates and incubated at 37°C for 24 h. After incubation, the media in each well was carefully aspirated to not disrupt the biofilms and the plates washed with 100 *μ*L PBS (thrice) to remove nonadherent and/or planktonic cells that remained in the wells. Afterwards, 100 *μ*L of XTT/menadione reagent was added to the wells and the plates incubated at 37°C for 2 h in the dark. Thereafter, 80 *μ*L of the resulting-coloured supernatant from the wells was transferred into new microplates and the plates measured spectrophotometrically at 490 nm [42].

#### 2.5.2. Inhibition of Preformed Biofilm

Fungal suspension (100 *μ*L of 1 × 10^6^ cells/mL in RPMI 1640) of each *C. albicans* strain was transferred into wells of a 96-well plate and incubated for 24 h at 37°C to allow for biofilm formation. The media from the wells were then aspirated carefully so not to touch the biofilms formed and washed twice with 100 *μ*L PBS to remove nonadherent and/or planktonic cells.

Dilutions of SBL and SBB were prepared from 1500 to 5.85 *μ*g/mL in another 96-well plate and added to the well plates that had the preformed biofilms. This was further incubated for 24 h at 37°C. Afterwards, the media in the wells were aspirated carefully and the plate washed two times with 100 *μ*L PBS. XTT/menadione solution (100 *μ*L) was added to each well, and the plates were incubated at 37°C for 2 h in the dark. Afterwards, 80 *μ*L of the resulting supernatant from the wells was transferred into a new microplate which was measured at 490 nm on a microplate reader [43].

Percentage inhibitions in both assays were determined as

(1) $$\begin{array}{c} \%\text{Inhibition}=\frac{\text{Absorbance of control}–\text{Absorbance of treatment }}{\text{Absorbance of control}}\times 100. \end{array}$$

The absorbances were analyzed with GraphPad for Windows version 8 (GraphPad Prism Software, San Diego, USA). The experiment was also replicated thrice in both assays.

### 2.6. Phytochemical Investigations

#### 2.6.1. Characterization of Extracts by UPLC-ESI-QTOF-MS/MS

The phytochemical characterization of SBL and SBB extracts was carried out following a procedure previously described in the literature with some modifications [44]. The separation was performed with a UHPLC Dionex Ultimate 3000 RS Liquid Chromatography System, on a C18 column (2.1 × 100 mm, 2.2 *μ*m) with a binary gradient (A: water with 0.1% formic acid; B: acetonitrile with 0.1% formic acid) at 0.4 mL/min at an injection volume of 2 *μ*L: from 0 to 0.4 min—isocratic at 5% B; 0.4 to 9.9 min—linear from 5% B to 100% B; 9.9 to 15.0 min—isocratic at 100% B; 15.0 to 15.1 min—linear from 100% B to 5% B; and 15.1 to 20.0 min—isocratic at 5% B.

The eluted compounds were detected with Dionex Ultimate DAD-3000 RS over wavelength of 200–400 nm and Bruker Daltonics micrOTOF-QII time-of-flight mass spectrometer with an Apollo electrospray ionisation source in a positive mode at 3 Hz over a mass range of *m*/*z* 50–1500 using the instrument settings: dry gas nitrogen, 9 L/min, 220°C; nebulizer gas nitrogen, 4 bar; capillary voltage, 4500 V; end plate offset, -500 V; transfer time, 100 *μ*s; collision gas nitrogen; collision energy; and collision RF settings were combined to each single spectrum of 1250 summations as follows: 624 summations with 80 eV collision energy and 130 Vpp + 313 summations with 16 eV collision energy and 130 Vpp + 313 summations with 16 eV collision energy and 130 Vpp. Internal dataset calibration (HPC mode) was done for each analysis with the mass spectrum of a 10 mM solution of sodium formate in 50% isopropanol that was infused during LC reequilibration using a diverter valve equipped with a 20 *μ*L sample loop.

#### 2.6.2. UPLC Fingerprint Profiling of Plant Extracts

Fingerprint profiles of the 70% ethanol extracts of SBL and SBB were produced using from UPLC analysis with Acquity UPLC® (Waters, Milford, U.S.A.) system equipped with PDA e*λ* detector (200-400 nm); QDa detector (ESI, positive mode, single quadrupole, 100–600 Da); sample manager (inj.-vol.: 2 *μ*L); column heater (40°C); stationary phase: Waters Acquity UPLC® HSS T3 (2.1 × 100 mm, 1.8 *μ*m); Empower 3 Software; a binary solvent manager with a flow rate: 0.5 mL/min, and mobile phases: A: H_2_O+ 0.1% formic acid, B: CH_3_CN+ 0.1% formic acid in a gradient elution format. With a run time of 13 minutes, the elution system of SBL was as follows: 0–1 min, 98%–90% A; 1–2 min, 90% A; 2–4 min 90%–85% A; 4–10 min, 85% A; 10–11 min, 85%-0% A; 11-12 min, 0%-98% A; and 12-13 min, 98% A. For SBB, the elution system was also as follows: 0–1 min, 0% A; 1–10 min, 0%-5% A; 10–15 min, 5% A; 15–16 min, 5%-10% A; and 16–22 min, 10% A. The run time for SBB was 22 minutes. The chromatograms of SBL and SBB extracts were recorded at 330 nm and 280 nm, respectively. The compounds present were then confirmed from the corresponding mass spectral data to the peaks observed. For qualitative purposes, the relative retention times (RRT) and relative peak areas (RPA) of the prominent UV-absorbing phytoconstituents observed in the chromatograms were calculated, in reference to internal standards, which also form part of the constituents of the extracts. Peaks corresponding to rutin and gallic acid in SBL and SBB, respectively, were designated as internal standards.

The fingerprints were then validated following the ICH Q2 (R1) guidelines [45]. The parameters considered included specificity, precision, and stability. Specificity was evaluated by identifying the key constituents in the extracts though their mass spectral data generated and spiking with reference compounds. Rutin and isoquercitrin were used as references for SBL extracts, whereas gallic acid and procyanidin B2 were used for SBB. Precision parameters, including repeatability and intermediate precision, were investigated by observing the RRTs and RPAs of five of the prominent peaks from replicate analysis of the extracts on same and different days. The relative standard deviations were then determined. The stability of the fingerprints was determined over a 48-hour period at predetermined time intervals (0, 6, 12, 24, and 48 hours). The percentage change in the relative peak areas of selected marker compounds (SBL–isoquercitrin; SBB–procyanidin B2) was then monitored.

## 3. Results

### 3.1. Antifungal Activity of *S. Birrea* Leaves and Stem Bark Extracts

The 70% ethanol leaf and stem bark extracts of *S. birrea* demonstrated moderate to strong antifungal activities against the *C. albicans* strains with MICs from 12.21 to 97.66 *μ*g/mL and MFCs 12.21 to 390.63 *μ*g/mL. The overall anti-*C. albicans* activity of the stem bark extract (SBB), however, was higher than the leaf extract (SBL) (Table 1). Except for the susceptible strains ATCC 90028 and SC5314, all the tested strains of *C. albicans* showed resistance to fluconazole. Voriconazole demonstrated variable inhibitory activities on the *C. albicans* strains (MIC = 4–16 *μ*g/mL).

### 3.2. Effect of *S. birrea* Leaf and Stem Bark Extracts on the Antifungal Activity of Conventional Antifungal Agents

The activity of fluconazole, nystatin, and caspofungin in combination with SBL or SBB against the *C. albicans* strains was investigated in the checkerboard assay. As seen in Table 2, after determining the respective FICIs, majority of the combinations of the extracts with the conventional antifungals showed indifference with occasional demonstration of synergism. Generally, SBL exhibited more synergistic interactions with the antifungal agents than SBB. However, SBB demonstrated more synergistic interactions with nystatin against the *C. albicans* strains.

### 3.3. Effect of *S. birrea* Leaf and Stem Bark Extracts on the Biofilms of *C. albicans* Strains

The activity of *S. birrea* leaf and stem bark hydroethanolic extracts against the biofilms of the *C. albicans* strains was examined under two experimental modalities. The first assay evaluated the tendency of the extracts to inhibit biofilm formation whereas the second assessed the activity of the extracts against preformed biofilms. As observed in Table 3, the preformed biofilms were more resistant to the extracts compared to the planktonic cells recording IC_50_ ranging from 91.50 to 685.20 *μ*g/mL. On the other hand, SBL and SBB demonstrated strong antibiofilm formation activity with IC_50_ from 12.49 to 164.42 *μ*g/mL. Generally, the SBB demonstrated higher activity against the biofilms of the *C. albicans* strains than SBL.

### 3.4. Phytochemical Characterization of the Extracts

**Figure 1:**
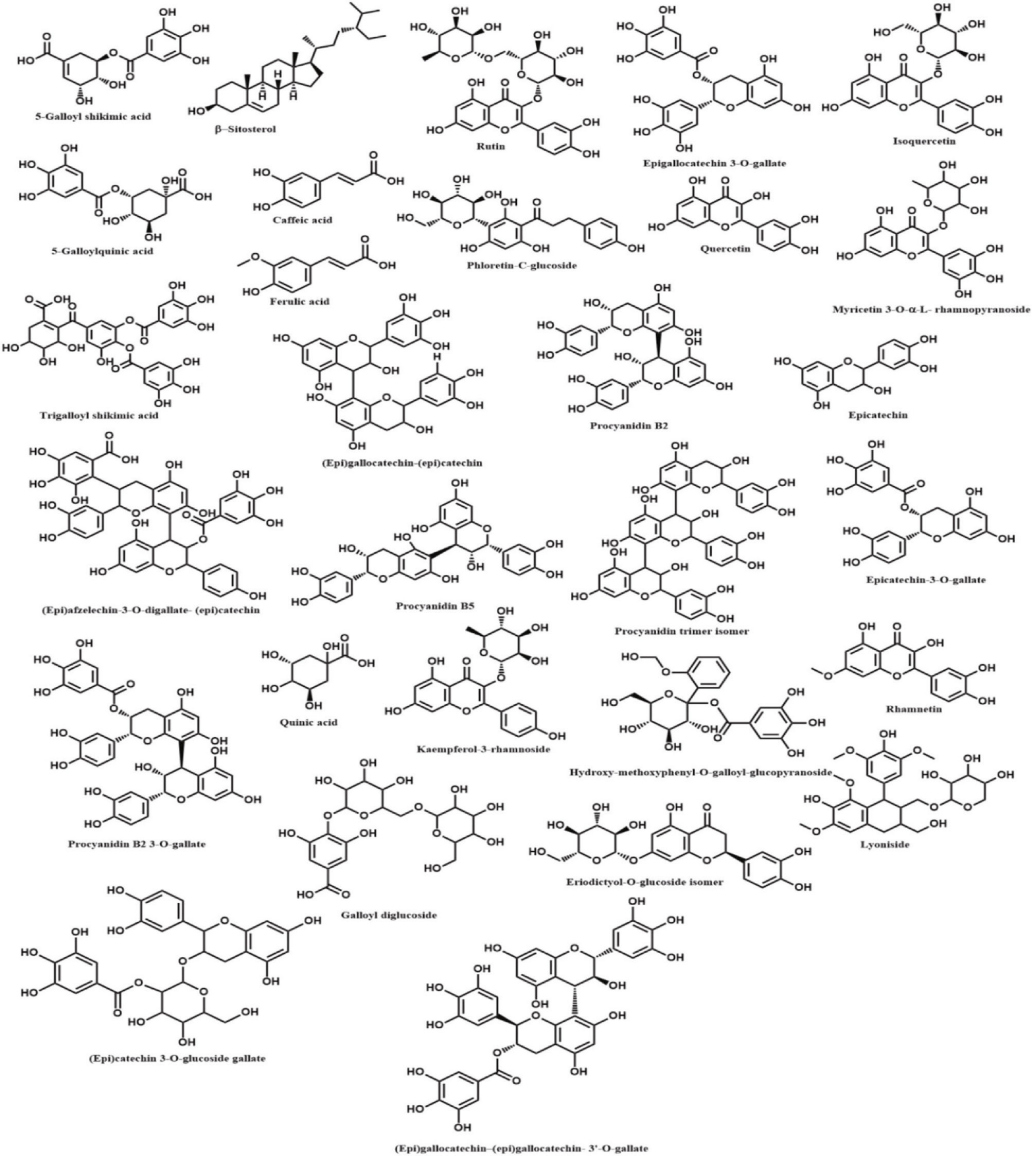
Chemical structures of phytochemical compounds identified in the hydroethanolic extracts of the leaves and stem bark of *S. birrea*.

The analysis shows the predominance of simple phenolic and polyphenolic compounds in both leaves and stem bark of the plants (Figure 1). SBL was observed to contain flavonoids like rutin, quercetin, isoquercetin, myricetin 3-O-*α*-L-rhamnopyranoside, and phloretin-c-glucoside. Caffeic acid, ferulic acid, galloylated quinic acid, and dimers of catechins and/or epicatechins were also observed. In the SBB, epicatechin monomers with their condensed tannins, including procyanidin B2, procyanidin C trimer isomer, and procyanidin B2 3-O-gallate, were detected in addition to the flavonoids and others. The phytochemical profiles of ethanolic extracts of SBL and SBB are shown in the supplementary data.

### 3.5. Fingerprint Profiling of SBL and SBB

**Figure 2:**
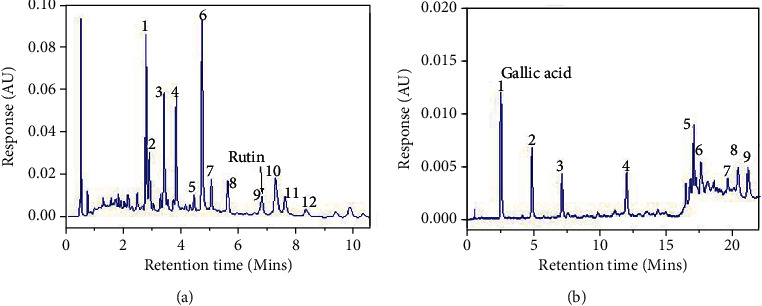
Representative UPLC fingerprint profiles for SBL (a) and SBB (b) extracts of *S. birrea*. The fingerprints show the prominent peaks used to identify the extracts together with their internal reference markers. In SBL, the following compounds were identified: galloyl shikimic acid [2], hydroxy-methoxyphenyl-O-galloyl-glucopyranoside [3], myricetin 3-O-*α*-L-rhamnopyranoside [8], rutin [9], isoquercetin [10], and quercetin 3-O-*α*-L-rhamnopyranoside [11]. In the SBB extract, the compounds identified included gallic acid [1], procyanidin B2 [4], procyanidin B5 [5], procyanidin B2 3-O-gallate [7], and procyanidin C trimer isomer [8].

The fingerprints of SBL and SBB are shown in Figure 2. SBL fingerprint showed the presence of flavonoids like rutin, isoquercitrin, and myricetin-3-O-*α*-L-rhamnopyranoside among others. In the fingerprint of SBB, however, gallic acid and dimers and trimers of condensed tannins like procyanidin B2, B5, and C trimer were observed. The identities of these compounds present as peaks in the fingerprint were confirmed from their retention times and their associated UV and mass spectra. The RRTs and RPAs (Table 4) were shown to be precise from both repeatability and intermediate precision analyses (Table 5). The RPAs were also stable beyond 48 hours of standing of the test solutions. These outcomes indicate that the fingerprints developed and validated were suitable preliminary qualitative tools to authenticate the plant and its parts for medicinal use.

## 4. Discussion

The current study investigated the antifungal potential of the hydroethanolic extracts of *S. birrea* leaves (SBL) and stem bark (SBB) against fluconazole-resistant *C. albicans* strains and clinical isolates recovered from pregnant women with vulvovaginal candidiasis.

SBB demonstrated strong antifungal activity (MIC = 12.21–48.83 *μ*g/mL) against the *C. albicans* strains whereas SBL exhibited strong to moderate activity (MIC = 12.21–97.66 *μ*g/mL). The MFCs of the extracts against the same set of strains were also evaluated. As observed from Table 1, higher concentrations of the extracts (MFC = 48.83 to 390.63 *μ*g/mL) are needed to exert fungicidal activity against the *C. albicans* strains and isolates. The results, however, are noteworthy as the emergence of resistance has become a significant clinical challenge that limits the successful treatment of *Candida* infections. Indeed, there has been an increase in the number of patients suffering from recurrent VVC in the last few decades which is attributable to the reduced sensitivity of *C. albicans* to the azole antifungals [46]. The observed activity of the leaves and stem bark of *S. birrea* against the fluconazole-resistant strains and isolates, which is being reported for the first time in this study, suggests that the leaves and stem bark of *S. birrea* are a promising alternative for treating *C. albicans* infections including those that may fail standard therapy and are potentially great sources of bioactive compounds that can be developed into novel antifungal agents that may overcome the challenge of resistance.

The low MICs recorded for the extracts suggests that they could be used together with conventional antifungals for the development of combinatorial therapies. The advantage being that these plant extracts if used together with current available antifungals, both at lower doses, could result in increased activity coupled with a decrease in their adverse effects. Studies have also shown that plant extracts, particularly those with antifungal activities, require high concentrations to be effective which might prove toxic to human health [47]. Additionally, combining antifungals with different mechanisms of action has been shown to be an innovative approach to overcoming the spread of fungal resistance [48]. Hence, we investigated the antifungal combination activity of SBL and SBB with fluconazole, nystatin, and caspofungin, as representatives of each class of antifungal agents: azoles, polyenes, and echinocandins, by the broth microdilution checkerboard assay. Investigations of the combination of SBL with the antifungals yielded mixed results, depending on the antifungal and the *C. albicans* strain or isolate tested. The predominant interaction observed in the *C. albicans* strains was indifference; however, synergism was also observed in some of the *C albicans* isolates. On the other hand, majority of combinations of SBB with the antifungal drugs demonstrated indifference, with the prominent exemption of the combinations with nystatin, which resulted in synergism in 75% of the tested strains and isolates. It has been hypothesized that synergism in antifungal combinations is expected when the evaluated antifungals have uniquely different mechanisms of action [41]. Thus, SBB may contain compounds which possess a mechanism of antifungal action different from nystatin, which is the disruption of the cell membrane via the binding to ergosterol in the fungal cell wall and the subsequent changes in cell permeability [49]. SBB, as shown from the phytochemical investigations, contained mostly proanthocyanidins, their galloylated derivatives, and flavonoids (Table S2) as compared to predominance of flavonoids in SBL. Flavonoids, such as rutin, quercetin, myricetin, kaempferol, and epigallocatechin gallate among others, have been shown to be effective against *Candida* species [50]. They are reported to exert this effect by inhibiting efflux pumps and inducing apoptosis, damaging cell walls, and disrupting cell membrane [51]. Similarly, proanthocyanidins and their galloylated derivatives also possess antifungal effects against Candida species [52], by inhibiting NF-кB p65 activation and subsequent phosphorylation of specific signal intracellular kinases [53]. The relatively different mechanisms by which these compounds exert their effect as compared to the conventional drugs may contribute to the synergistic effects observed in some instances. Also, the presence of the proanthocyanidins and their galloylated derivatives in addition to the flavonoids in SBB could be thought to contribute to the differences in activities of the two plant parts. Overall, these results show that SBL and SBB could be used in combination with conventional antifungal drugs.

The ability to form biofilms is critical to the pathogenicity and the development of fungal resistance which can decrease the sensitivity of *C. albicans* to currently used antifungals up to a thousand times than planktonic cells [54]. Therefore, the search for anti-*C albicans* biofilms has become a pressing need. The activity of SBL and SBB against the preformed biofilms as well as their tendencies to inhibit biofilm formation of the *C. albicans* strains and isolates was evaluated in the study. As expected, greater concentrations (3–14 folds) of the extracts were needed to inhibit the preformed biofilms than those required to inhibit biofilm formation. Although this is the first report of the activity of *S. birrea* against *Candida* biofilms, the plant had previously been shown to exert antibiofilm activity in bacteria. The methanol extract of *S. birrea* stem bark demonstrated a concentration-dependent antibiofilm formation activity in *Pseudomonas aeruginosa* with a maximum inhibition of 87.45% at 200 *μ*g/mL [55]. The effects against the biofilm formation as well as disruption of preformed biofilms were also seen to be better in SBB than SBL, and this could also be because of the proanthocyanidins present [52, 53].

The observed antifungal activities of *S. birrea* may therefore be because of flavonoids and proanthocyanidins and their derivatives present in SBL and SBB working synergistically, additively, and/or in a potentiation manner to contribute to the overall activity of the plant.

Whereas Hamza et al. [33] demonstrated that the root bark of the plant has antifungal activity against susceptible strains of *Candida* species, we, in this study, have shown that the leaves and stem bark of the plant possess antifungal action against resistant strains and isolates of *C. albicans*, the major causative agent of candidiasis. The use of the leaves and stem bark will promote sustainable use of the plant and would therefore recommend their usage rather than the roots, in traditional preparations and in the herbal industry to conserve the plant.

The proposed analytical conditions reported herein are suitable for the preliminary quality assessment of the leaves and stem bark of the plant either for traditional use or their use in herbal medicine manufacture. While the RRTs provide valuable qualitative information to assess the presence of the annotated peaks in the extracts, their RPAs provide semiquantitative information which can be used to estimate the contents of the respective peaks [56]. The outcome of the validation also indicates the reliability of the fingerprints to generate reproducible information. The UPLC fingerprints developed and validated can therefore be used in their entirety or by way of verifiable constituent marker compounds (using their RRTs and RPAs) to verify the authenticity of a sample of either of the parts investigated. However, more UPLC fingerprints should be developed from 70% ethanol extracts of the leaves and stem bark of *S. birrea* collected from different geographical location and seasons to further confirm the results.

## 5. Conclusion

We report, for the first time, the antifungal activity of the leaves and stem bark hydroethanolic extracts of *S. birrea* against fluconazole-resistant strains and isolates of *C. albicans*. The extracts demonstrated considerable antifungal activity and significantly inhibited biofilm formation activity of the *C. albicans* strains and isolates. They also inhibited considerably the activity of preformed biofilms of the resistant strains and isolates and demonstrated potential for their use as combination therapies with currently used antifungals especially the stem bark extract with nystatin. These effects may be due to the presence of flavonoids, proanthocyanidins, and their galloylated derivatives confirmed in the extracts. Additionally, analytical conditions have been proposed for UPLC fingerprint profiling, and this could be used to verify the authenticity of the leaves and stem barks of *S. birrea*.

## Figures and Tables

**Table 1:** Inhibitory effect of *S. birrea* leaf and stem bark extracts on *C. albicans* strains.

Strain	SBL		SBB		FLC	VRC
	MIC	MFC	MIC	MFC	MIC	MIC
ATCC 90028	12.21	12.21	12.21	12.21	4.00	4.00
ATCC 10231	97.66	390.63	24.41	48.83	—	16.00
SC5314	24.41	195.31	12.21	48.83	8.00	4.00
CA-R1	48.83	195.31	24.41	48.83	—	8.00
CA-R2	48.83	195.31	24.41	48.83	—	16.00
CA-R3	97.66	390.63	24.41	97.66	—	16.00
CA-R4	48.83	195.31	24.41	48.83	—	8.00
CA-R5	97.66	195.31	48.83	195.31	—	16.00

Values are in *μ*g/mL (*n* = 3). SBL: *S. birrea* 70% ethanol leaf extract; SBB: *S. birrea* 70% ethanol stem bark extract; MIC: Minimum Inhibitory Concentration; MFC: Minimum Fungicidal Concentration; FLC: fluconazole; VRC: voriconazole.

**Table 2:** Effect of *S. birrea* leaf and stem bark extracts in combination with fluconazole, nystatin, or caspofungin against *C. albicans* strains.

Strains	Combinations											
	SBL						SBB					
	FLC		NYS		CSF		FLC		NYS		CSF	
	FICI	INT	FICI	INT	FICI	INT	FICI	INT	FICI	INT	FICI	INT
ATCC 90028	0.50	**S**	0.5	**S**	0.28	**S**	1.50	**I**	0.38	**S**	0.63	**I**
ATCC 10231	0.31	**S**	0.38	**S**	0.75	**I**	1.50	**I**	0.28	**S**	0.38	**S**
SC5314	2.00	**I**	1.00	**I**	1.13	**I**	1.50	**I**	0.28	**S**	2.13	**I**
CA-R1	2.00	**I**	2.00	**I**	0.75	**I**	2.00	**I**	1.25	**I**	2.13	**I**
CA-R2	0.31	**S**	1.50	**I**	4.25	**A**	2.00	**I**	1.25	**I**	4.06	**A**
CA-R3	0.75	**I**	1.25	**I**	2.00	**I**	2.00	**I**	0.50	**S**	2.13	**I**
CA-R4	0.75	**I**	0.02	**S**	3.00	**I**	1.50	**I**	0.5	**S**	4.06	**A**
CA-R5	0.02	**S**	0.02	**S**	1.50	**I**	1.50	**I**	0.5	**S**	2.13	**I**

FLC: fluconazole; NYS: nystatin; CSF: caspofungin. FICI: Fraction Inhibitory Concentration Index; S: synergism for FICI ≤ 0.5; I: indifference FICI was >0.5 to ≤4.0; A: antagonism FICI >4.0 (*n* = 3).

**Table 3:** Inhibitory effect of *S. birrea* leaf and stem bark extracts on biofilm formation and preformed biofilms of *C. albicans* strains.

Strains	Inhibition (IC_50_)			
	Biofilm formation		Preformed biofilm	
	SBL	SBB	SBL	SBB
ATCC 90028	100.30	12.49	223.00	91.50
ATCC 10231	164.42	75.12	543.60	522.40
SC5314	108.40	17.85	303.00	93.82
CA-R1	180.23	23.80	249.60	127.90
CA-R2	120.90	28.86	363.50	105.10
CA-R3	116.00	24.40	470.30	245.60
CA-R4	118.70	39.20	685.20	540.80
CA-R5	102.70	30.04	430.90	315.80

Values are in *μ*g/mL (*n* = 3). SBL: *S. birrea* 70% ethanol leaf extract; SBB: *S. birrea* 70% ethanol stem bark extract.

**Table 4:** Relative retention times (RRT) and relative peak areas (RPA) for *S. birrea* plant extracts as determined for qualitative purposes.

Peak number^∗^	SBL			SBB		
	Retention time (min)	Relative retention time	Relative peak area	Retention time (mins)	Relative retention time	Relative peak area
1*α*	2.77	0.41	5.1937	2.53	1.00	1.0000
2	2.89	0.42	1.8516	4.87	1.93	0.4560
3	3.40	0.50	3.2967	7.11	2.81	0.3229
4	3.82	0.56	2.8892	11.98	4.74	0.4001
5	4.45	0.65	0.4490	17.01	6.73	0.2742
6	4.73	0.69	5.7176	17.57	6.95	0.3395
7	5.05	0.74	0.9624	19.57	7.74	0.0786
8	5.61	0.82	1.2910	20.37	8.06	0.2572
9*β*	6.81	1.00	1.0000	21.07	8.33	0.3385
10	7.28	1.07	2.2503			
11	7.62	1.12	0.9235			
12	8.33	1.22	0.5426			

^∗^Peak numbers used to illustrate the compounds in the chromatograms for SBL and SBB do not necessarily correspond to similar compounds in the two extracts. *α*Compound 1 identified as gallic acid was used as an internal reference marker for SBB extract. *β*Compound 9 identified as rutin was used as an internal reference marker for SBL extract.

**Table 5:** Validation parameters for UPLC fingerprint profiling of SBL and SBB samples.

Extract	Peak no.	Precision				Stability (over 48 hours) *Δ*RPA (%)
		Repeatability (same day) (*n* = 6)		Intermediate precision (different days) (*n* = 9)		
		Mean RRT, RSD (%)	Mean RRA, RSD (%)	Mean RRT, RSD (%)	Mean RRA, RSD (%)	
SBL	1	0.41 ± 0.002, 0.42	5.192 ± 0.037, 0.71	0.41 ± 0.006, 1.56	5.185 ± 0.041, 0.79	Peak 4–procyanidin B2: 3.39
	2	0.50 ± 0.001, 0.25	3.306 ± 0.037, 1.13	0.50 ± 0.016, 3.15	3.296 ± 0.040, 1.20	
	3	0.56 ± 0.002, 0.31	2.890 ± 0.022, 0.75	0.56 ± 0.007, 1.25	2.903 ± 0.026, 0.90	
	4	0.69 ± 0.001, 0.16	5.724 ± 0.017, 0.30	0.69 ± 0.008, 1.20	5.725 ± 0.024, 0.42	
	5^∗^	1.00	1.00	1.00	1.00	

SBB	1^∗^	1.00	1.00	1.00	1.00	Peak 1–gallic acid: 1.74
	2	1.93 ± 0.003, 0.17	0.456 ± 0.003, 0.68	1.92 ± 0.019, 0.98	0.456 ± 0.007, 1.64	
	3	2.81 ± 0.004, 0.14	0.323 ± 0.005, 1.48	2.88 ± 0.057, 1.99	0.322 ± 0.005, 1.62	
	4	4.73 ± 0.005, 0.11	0.402 ± 0.003, 0.76	4.74 ± 0.033, 0.70	0.4000 ± 0.002, 0.61	
	5	6.72 ± 0.002, 0.03	0.276 ± 0.001, 0.35	6.74 ± 0.035, 0.52	0.276 ± 0.002, 0.54	

Acceptance criteria		**R** **S** **D** < 2%	**R** **S** **D** < 2%	**R** **S** **D** < 2%	**R** **S** **D** < 2%	**%**Δ**RPA** < 5%

^∗^Internal reference marker compound in the respective extracts of the plant.

## Data Availability

All available data are included in the manuscript and supplementary data.

## Supplementary Materials

Supplementary Materials The metabolite profiles of the 70% ethanol extracts of *S. birrea* leaves and stem bark are presented in the supplementary data.
