# Supplementary material for: Antifungal Activities of Phytochemically Characterized Hydroethanolic Extracts of Sclerocarya birrea Leaves and Stem Bark against Fluconazole-Resistant Candida albicans Strains
Source: Biomed Res Int. 2022 Jun 7;2022:4261741. doi: 10.1155/2022/4261741 (PMC9197620; doi:10.1155/2022/4261741)
Supplement: Supplementary Materials — The metabolite profiles of the 70% ethanol extracts of S. birrea leaves and stem bark are presented in the supplementary data. [file 4261741.f1.docx]

**Antifungal Activities of Phytochemically Characterized Hydroethanolic Extracts of *Sclerocarya birrea* Leaves and Stem bark against Fluconazole-Resistant *Candida albicans* strains**

Benjamin Kingsley Harley ^1*^, David Neglo^2^, Mike Okweesi Aggrey^1^, Anthony Martin Quagraine^1^, Emmanuel Orman^3^, Jonathan Jato^1^, Nana Ama Mireku-Gyimah^4^, Cedric Dzidzor Amengor^3^, Theophilus Christian Fleischer^1^

^1^Department of Pharmacognosy and Herbal Medicine, School of Pharmacy, University of Health and Allied Sciences, Ho, Ghana.

^2^Department of Basic Science, School of Basic and Biomedical Sciences, University of Health and Allied Sciences, Ho, Ghana.

^3^Department of Pharmaceutical Chemistry, School of Pharmacy, University of Health and Allied Sciences, Ho, Ghana.

^4^Department of Pharmacognosy and Herbal Medicine, School of Pharmacy, University of Ghana, Accra, Ghana

***Corresponding Author**

Name: Benjamin Kingsley Harley

Address: Department of Pharmacognosy and Herbal Medicine

School of Pharmacy

University of Health and Allied Sciences

P. O. Box 31

Ho- Volta Region

Ghana

Phone number: +233 265398208

E-mail address: [bkharley@uhas.edu.gh](mailto:bkharley@uhas.edu.gh)


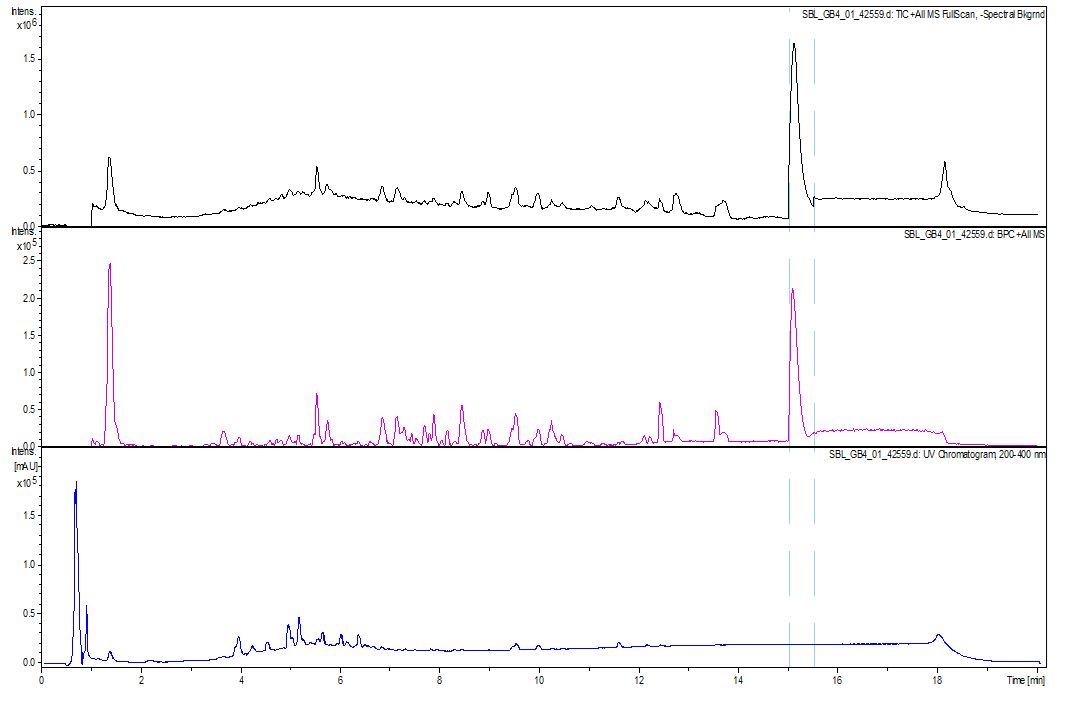


**Figure S1:** Stack layout of Total Ion, Base Peak and UV chromatograms of 70% ethanol leaves extract of *S. birrea* from the UPLC-ESI-QTOF-MS/MS Analysis.


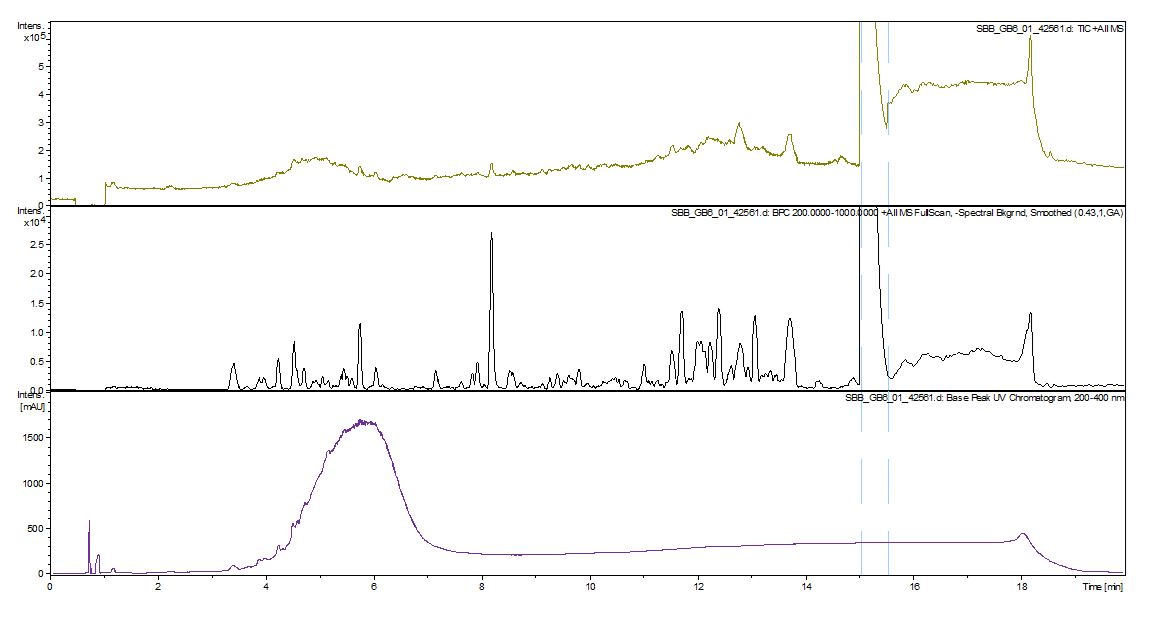


**Figure S2:** Stack layout of Total Ion, Base Peak and UV chromatograms of the 70% ethanol stem bark extract of *S. birrea* from the UPLC-ESI-QTOF-MS/MS Analysis.

**Table S1:** Proposed compounds identified in the leaves of *S. birrea* from UPLC-ESI-QTOF-MS/MS analysis

| S/N | Retention time (min) | | Observed m/z [Adduct(s)] | Monoisotopic mass | MS/MS Fragments | Accuracy (ppm/mDa) | Molecular Formula | Identification | Reference |
| --- | --- | --- | --- | --- | --- | --- | --- | --- | --- |
| 1 | 3.96 | 349.09 (M+Na) | | 326.10 | 147.04 (+1) | 4 mDa | C_14_H_14_O_9_ | 5-Galloyl shikimic acid | [1] |
| 2 | 4.66 | 437.23 (M+Na) | | 414.24 |  | 10 ppm | C_29_H_50_O | β-sitosterol |  |
| 3 | 4.83 | 481.25 (M+Na) | | 458.26 |  | 20 ppm | C_22_H_18_O_11_ | Epigallocatechin 3-O-gallate | [2, 3] |
| 4 | 5.02 | 611.16 (M+H) | | 610.16 |  | 10 ppm | C_27_H_30_O_16_ | Rutin | [1] |
| 5 | 5.16 | 465.10 (M+H) | | 464.10 | 325.10 (+1); 303.05 (+1) | 10 ppm | C_21_H_20_O_12_ | Isoquercetin | [4] |
| 6 | 5.34 | 465.20 (M+H) | | 464.19 |  | 20 ppm | C_21_H_20_O_12_ | Myricetin 3-O-α-L- rhamnopyranoside | [4] |
| 7 | 6.29 | 345.10 (M+H) | | 344.10 |  | 4 mDa | C_14_H_16_O_10_ | 5-Galloylquinic acid | [1] |
| 8 | 7.71 | 203.10 (M+Na) | | 180.11 |  | 4 mDa | C_9_H_8_O_4_ | Caffeic acid | [5] |
| 9 | 7.95 | 437.16 (M+H) | | 436.15 | 349.11 (+1); 295.06 (+1) | 10 ppm | C_21_H_24_O_10_ | Phloretin-C-glucoside | [1] |
| 10 | 11.54 | 217.11 (M+H) | | 194.12 |  | 8 mDa | C_10_H_10_O_4_ | Ferulic acid | [5] |
| 11 | 12.16 | 325.21 (M+Na) | | 302.22 |  | 8 mDa | C_15_H_10_O_7_ | Quercetin |  |
| 12 | 13.05 | 333.16 (M+H) | | 332.16 |  | 8 mDa | C_13_H_16_O_10_ | Galloyl glucoside isomer | [1] |
| 13 | 13.05 | 653.49 (M+Na) | | 630.51 |  | 20 ppm | C_28_H_23_O_17_ | Trigalloyl shikimic acid | [1] |
| 14 | 13.17 | 617.47 (M+Na) | | 594.48 |  | 10 ppm | C_30_H_26_O_13_ | (Epi)gallocatechin-(epi)catechin isomer | [1] |
| 15 | 14.43 | 485.38 (M+Na) | | 462.39 |  | 10 ppm |  | Pentoside derivative | [2] |

**Table S2:** Proposed compounds identified in the stem bark of *S. birrea* from UPLC-ESI-QTOF-MS/MS analysis

| S/N | Retention time (min) | | Observed m/z [Adduct(s)] | Monoisotopic mass | MS/MS Fragments | Accuracy (ppm/mDa) | Molecular Formula | Identification | Reference |
| --- | --- | --- | --- | --- | --- | --- | --- | --- | --- |
| 1 | 3.40 | 579.16 (M+H) | | 578.15 | 289.09(+1); 427.11 (+1) | 10 ppm | C_30_H_26_O_12_ | Procyanidin B2 | [2] |
| 2 | 3.91 | 291.09 (M+H) | | 290.08 | - | 8 mDa | C_15_H_14_O_6_ | Epicatechin | [2] |
| 3 | 3.96 | 867.22 (M+H) | | 866.21 | 289.08 (+1); 579.15 (+1) | 10 ppm | C_45_H_38_O_18_ | Procyanidin C trimer isomer |  |
| 4 | 4.23 | 579.15 (M+H) | | 578.15 | 289.08(+1); 427.11 (+1) | 10 ppm | C_30_H_26_O_12_ | Procyanidin B5 | [2] |
| 5 | 4.57 | 867.22 (M+H) | | 866.21 | 579.15 (+1); 163.04 (+1) | 10 ppm | C_44_H_34_O_19_ | (Epi)afzelechin-3-O-digallate- (epi)catechin | [1] |
| 6 | 4.71 | 731.16 (M+H) | | 730.16 | 289.07 (+1); 579.15 (+1) | 10 ppm | C_37_H_30_O_16_ | Procyanidin B2 3-O-gallate | [4] |
| 7 | 5.15 | 443.10 (M+H) | | 442.10 | 273.08 (+1); 139.04 (+1) | 10 ppm | C_22_H_18_O_10_ | Epicatechin-3-O-gallate | [2, 3] |
| 8 | 5.74 | 487.19 (M+Na) | | 464.20 | 435.20 (+1); 361.17 (+1) | 10 ppm | C_21_H_20_O_12_ | Myricetin 3-O-α-L- rhamnopyranoside | [2] |
| 9 | 6.16 | 193.08 (M+H) | | 192.08 | 175.08 (+1) | 4 mDa | C_7_H_12_O_6_ | Quinic acid | [2, 3] |
| 10 | 8.57 | 433.35 (M+H) | | 432.35 | - | 20 ppm | C_21_H_20_O_10_ | Kaempferol-3-rhamnoside | [2] |
| 11 | 8.57 | 455.34 (M+H) | | 454.33 | - | 10 ppm | C_20_H_22_O_12_ | Hydroxy-methoxyphenyl-O-galloyl-glucopyranoside | [2] |
| 12 | 9.66 | 317.24 (M+H) | | 316.24 |  | 8 mDa | C_16_H_12_O_7_ | Rhamnetin | [2] |
| 13 | 11.43 | 517.38 (M+Na) | | 494.39 | 261.13 (+1); 177.06 (+1) | 20 ppm | C_19_H_26_O_15_ | Galloyl diglucoside isomer | [1] |
| 14 | 11.46 | 473.35 (M+Na) | | 450.36 |  | 10 ppm | C_21_H_22_O_11_ | Eriodictyol-O-glucoside isomer | [2] |
| 15 | 11.46 | 512.42 (M+NH_4_) | | 494.39 |  | 20 ppm | C_19_H_26_O_15_ | Galloyl diglucoside isomer | [1] |
| 16 | 11.83 | 575.42 (M+Na) | | 552.43 |  | 20 ppm | C_27_H_36_O_12_ | Lyoniside | [2] |
| 17 | 11.83 | 605.41 (M + H) | | 604.41 |  | 20 ppm | C_28_H_28_O_15_ | (Epi)catechin 3-O-glucoside gallate | [2] |
| 18 | 13.17 | 617.47 (M+Na) | | 594.48 |  | 10 ppm | C_30_H_26_O_13_ | (Epi)gallocatechin-(epi)catechin isomer | [1] |
| 19 | 13.17 | 762.56 (M^+^) | | 762.56 |  | 20 ppm | C_37_H_30_O_18_ | (Epi)gallocatechin–(epi)gallocatechin- 3'-O-gallate | [1, 2] |
| 20 | 13.46 | 485.39 (M+H) | | 484.38 |  | 10 ppm | C_20_H_20_O_14_ | Digalloyl glucoside isomer | [1] |
| 21 | 13.59 | 458.35 (M^+^) | | 458.35 | - | 20 ppm | C_22_H_18_O_11_ | Epigallocatechin 3-gallate | [2, 3] |
